# Wilson’s Disease in an Early Adolescent: Classic Magnetic Resonance Imaging Findings

**DOI:** 10.7759/cureus.58092

**Published:** 2024-04-12

**Authors:** Praveen K Sharma, Arun Aram, Vinoth Pandian, Yashaswinii Polaka

**Affiliations:** 1 Department of Radiology, Saveetha Medical College and Hospital, Saveetha Institute of Medical and Technical Sciences, Saveetha University, Chennai, IND

**Keywords:** liver cirrhosis, magnetic resonance imaging, basal ganglia, copper, hepatolenticular degeneration

## Abstract

Wilson’s disease (WD), alternatively termed hepatolenticular degeneration, represents a rare autosomal recessive disorder typified by disrupted copper metabolism, culminating in copper accumulation across various organs. WD commonly manifests with early-onset liver cirrhosis, with notable involvement of the central nervous system, particularly impacting the midbrain and basal ganglia. This case report delineates the clinical presentation of an early adolescent female with WD, accentuating classical magnetic resonance imaging (MRI) findings. These MRI findings, which include the “face of a giant panda sign” and the “Face of a miniature panda sign,” are pivotal for expeditious diagnosis. Recognition of these classical signs underscores the indispensable role of MRI in elucidating the neurological dimensions of WD.

## Introduction

Wilson’s disease (WD) is a rare but medically significant condition characterized by its autosomal recessive inheritance. It disrupts normal hepatic copper transport, leading to an excessive accumulation of copper, primarily in the liver and central nervous system (CNS). This disorder typically emerges during adolescence, presenting a challenge for diagnosis due to its varied and multi-systemic manifestations [1]. In this report, we delve into a compelling case of WD in a young adolescent female, highlighting essential magnetic resonance imaging (MRI) findings. The genesis of WD can be traced back to mutations in the ATPase copper-transporting beta gene (*ATP7B *gene), which disrupt the balance of copper in the body [2]. This genetic defect interferes with the normal transport of copper in the liver, leading to an overload of copper that can cause significant damage to various organs, with the liver, brain, and cornea being particularly vulnerable [3]. The onset of WD symptoms in early adolescence underscores the importance of recognizing the diverse clinical manifestations to ensure prompt diagnosis and treatment. Our study emphasizes the significance of MRI findings in uncovering the neurological aspects of WD, shedding light on how the disease affects the CNS. Through a detailed case study, our objective is to contribute to the growing body of knowledge on WD, enhancing diagnostic precision and improving patient treatment outcomes.

## Case presentation

An 11-year-old Indian female patient, born of a consanguineous marriage, was brought to the pediatric clinic by her concerned parents due to a range of troubling symptoms that had progressively worsened over the past few months. Initially presenting with tremors, dystonia, dysarthria, and a noticeable yellowing of the skin and eyes indicative of jaundice, her condition raised immediate red flags for a deeper, systemic issue. Additionally, the patient had anxiety and cognitive impairment. Upon thorough medical evaluation, it was evident that the patient was suffering from significant hepatic dysfunction. This was characterized by hepatomegaly, and laboratory tests confirmed the suspicion with elevated liver enzyme levels, specifically alanine transaminase (ALT) at 120 U/L (normal range: 7 to 40 U/L) and aspartate transaminase (AST) at 135 U/L (normal range: 10 to 40 U/L), both markedly above the normal range. Additionally, total bilirubin was elevated at 2.5 mg/dL, with a direct bilirubin of 1.8 mg/dL, indicating cholestasis and hepatocellular injury. Further investigations were warranted to determine the underlying cause of her hepatic dysfunction. Neurologically, the patient’s tremors, dystonia, and dysarthria pointed toward CNS involvement, a hallmark of WD that often complicates the clinical picture due to its impact on motor coordination and speech. Given the symptoms and signs, WD was a significant consideration. Notably, despite the absence of a family history of liver disease, consanguineous marriage could be a relevant factor in this case.

**Figure 1:**
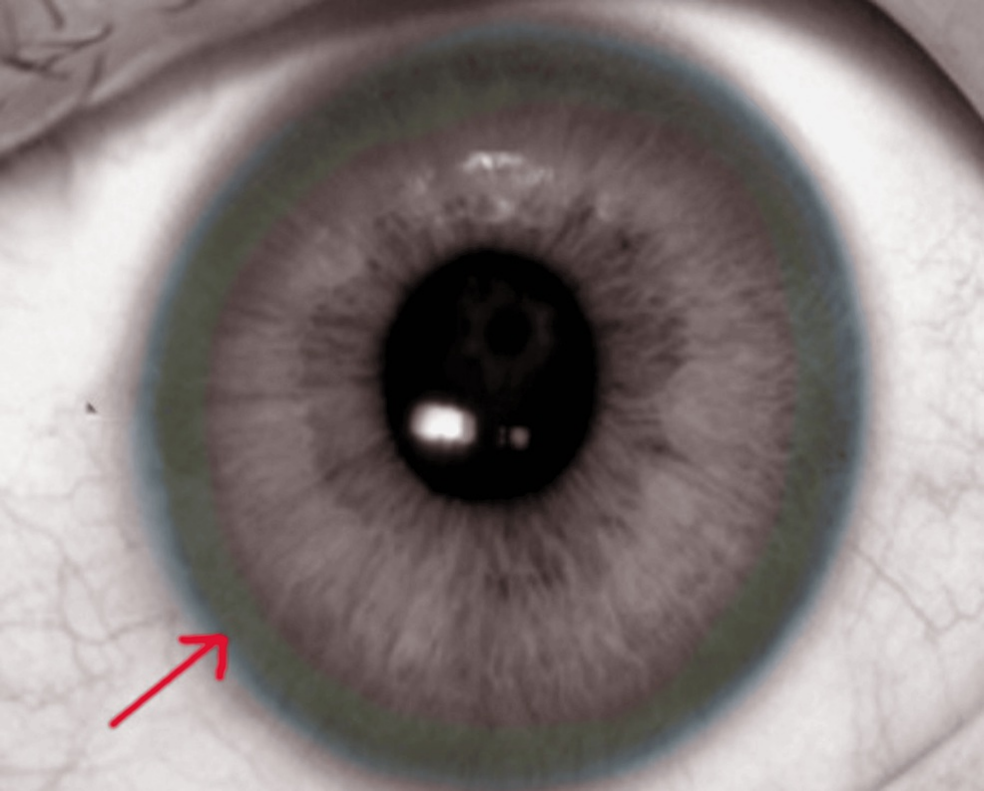
Kayser-Fleischer rings from copper deposits. The image shows the annular deposition of copper in the limbus of a patient’s cornea, also known as the Descemet membrane, which appears as a golden to greenish-brown ring. Contributed by O Chaigasame, MD.

The diagnostic workup was comprehensive and aimed at confirming WD, a rare autosomal recessive disorder marked by abnormal copper accumulation in the body. Serum ceruloplasmin levels were significantly reduced at 5 mg/dL (normal range: 20-35 mg/dL), a key indicator of WD, given ceruloplasmin’s role in copper transport. Urinary copper excretion was markedly elevated, with a 24-hour copper excretion rate of 200 µg/day (normal range: <100 µg/day), reinforcing the suspicion of WD. The results of liver function tests provided additional evidence of hepatic involvement. As ALT and AST levels alone may not serve as direct indicators of liver prognosis, a comprehensive evaluation was undertaken to elucidate both the underlying cause and the severity of hepatic dysfunction. An ophthalmological examination revealed the presence of Kayser-Fleischer rings, a distinctive sign of copper deposition in the cornea, offering critical diagnostic evidence for WD. These golden-brown or greenish rings encircling the iris are virtually pathognomonic for the disease, especially in neurological and hepatic symptoms. A representative image of Kayser-Fleischer rings is shown in Figure 1 [4].

**Figure 2:**
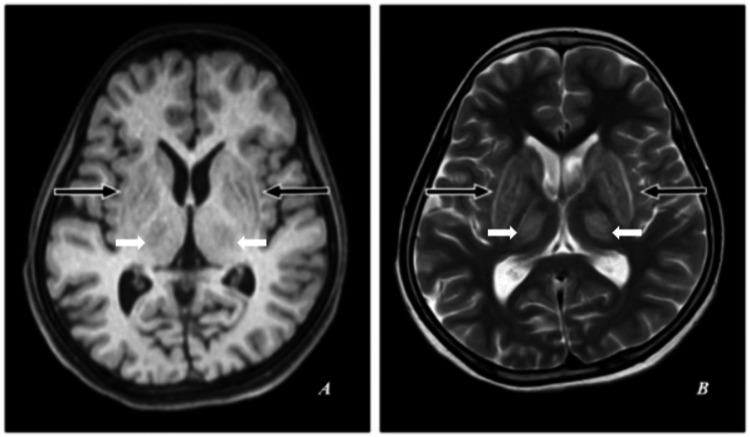
MRI of the brain. (A) T1-weighted (T1W) axial image. (B) T2-weighted (T2W) axial image. T1W: Symmetrical subtle hypointensities. T2W: Hyperintensities in the deep gray matter in the bilateral basal ganglia (lentiform nuclei: globus pallidus and putamen) and bilateral thalami (thick white arrows).

Symmetrical subtle hypointensities were observed on T1-weighted imaging, while T2-weighted imaging revealed hyperintensities in the deep gray matter in the bilateral basal ganglia (specifically the lentiform nuclei: globus pallidus and putamen), as well as in the bilateral thalami (Figure 2).

On T1-weighted imaging, a symmetrical thin rim of subtle hypointensities was evident, whereas on T2-weighted imaging, hyperintensities were observed in the sub-cortical gray matter in the bilateral claustrum, indicating the presence of the “bright claustrum sign.”

**Figure 3:**
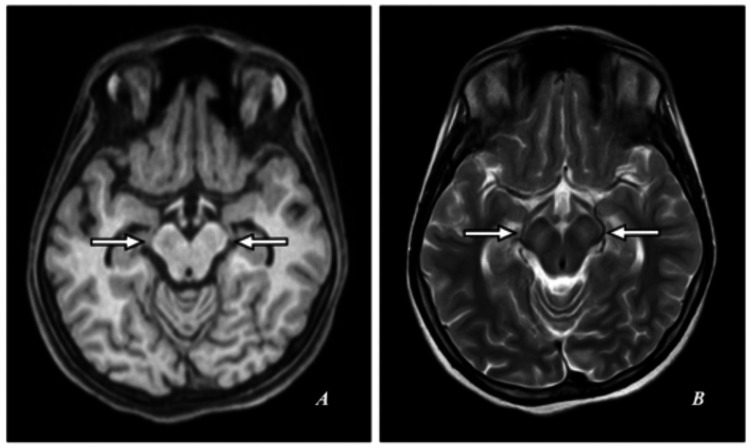
MRI of the brain. (A) T1-weighted (T1W) axial image. (B) T2-weighted (T2W) axial image. T1W: Symmetrical subtle hypointensities. T2W: Hyperintensities in the midbrain tegmentum with preservation of normal signal intensities of the lateral aspect of pars reticulata of substantia nigra, resembling the appearance of “ears,” normal signal intensities of the red nucleus, resembling the appearance of “eyes,” and hypointensity in the superior colliculus resembling the appearance of “chin,” showing the “face of a giant panda sign” (short white arrows).

**Figure 4:**
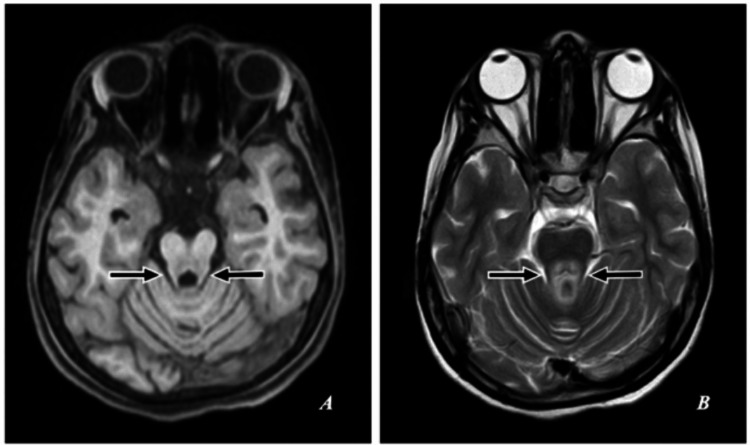
MRI of the brain. (A) T1-weighted (T1W) axial image. (B) T2-weighted (T2W) axial image. T1W: Symmetrical subtle hypointensities. T2W: Hypointensities in the pons of medial longitudinal fasciculus and central tegmental tract resembling the appearance of “eyes,” hyperintensity in the aqueduct opening into the fourth ventricle delineated at its base by the superior medullary velum resembling the appearance of “nose and mouth,” and hyperintensities in the superior cerebellar peduncles resembling the appearance of “cheek” showing the “face of a miniature panda sign” (short black arrows).

Subtle hypointensities were symmetrically observed on T1-weighted imaging. On T2-weighted imaging, hyperintensities appeared in the midbrain tegmentum, while normal signal intensities were retained in the lateral aspect of the pars reticulata of the substantia nigra, resembling “ears.” Furthermore, normal signal intensities resembling “eyes” were evident in the red nucleus, along with hypointensity in the superior colliculus, resembling a “chin,” indicative of the characteristic “face of a giant panda sign” (Figures 3, 4).

**Figure 5:**
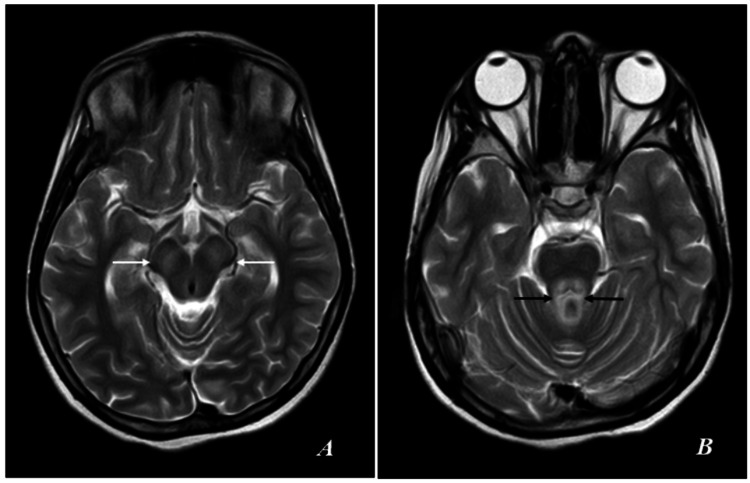
MRI of the brain. (A, B) T2-weighted axial image showing a combination of the “face of a giant panda sign” (short white arrows) and the “face of a miniature panda sign” (short black arrows) refers to the “double panda sign.”

The “double panda sign” illustrated in Figure 5 refers to the combination of both the “face of a giant panda sign” and the “face of a miniature panda sign.”

Diagnosis confirmation

The combination of clinical symptoms, laboratory abnormalities, ophthalmological findings, and classic MRI results collectively contributed to the diagnosis of WD in this early adolescent patient.

Management and follow-up

The patient was promptly initiated on chelation therapy with D-penicillamine, starting with an initial dose of 250 mg once daily for one month. The dosage was gradually increased to a maintenance dose of 20 mg/kg/day, given in two divided doses. This dosage regimen aimed to ensure effective copper removal while monitoring potential adverse effects such as hypersensitivity reactions, renal impairment, or neurological worsening. Regular follow-up was conducted to monitor the efficacy of treatment, and the dosage was adjusted as needed. During follow-up visits, assessments included liver function tests, neurological examination, and 24-hour urinary copper excretion to gauge the response to therapy. During follow-up visits, assessments, including liver function tests, neurological examination, and 24-hour urinary copper excretion, were performed to gauge the response to therapy. Treatment plans were adjusted accordingly based on these assessments. Additionally, a careful balance between minimizing side effects and maximizing therapeutic efficacy was maintained by monitoring for adverse effects of D-penicillamine such as skin reactions, renal toxicity, hepatotoxicity, and lupus-like syndrome [5]. Dietary modifications also played a supportive role in managing WD. Recommendations included avoiding copper-rich foods such as shellfish, nuts, chocolate, mushrooms, and organic meats. These dietary modifications further aided in controlling copper levels in the body. This integrated approach, combining D-penicillamine therapy with regular monitoring and dietary management, aimed to reduce copper accumulation, alleviate symptoms, and improve the quality of life for patients with WD. It underscores the importance of adhering to treatment protocols for effective long-term management.

## Discussion

This case report illuminates the significance of WD within the context of its diverse clinical manifestations, particularly highlighting its presentation in an early adolescent female. By emphasizing the variability in symptomatology and the need for considering WD in the diagnostic framework of hepatic and neurological disorders, the case underscores the importance of vigilance, especially in younger patients. Moreover, the discussion emphasizes the pivotal role of classical MRI findings, such as the “face of a giant panda sign” and the “face of a miniature panda sign,” in aiding diagnosis, particularly in cases with suspected neurological involvement. Furthermore, the comprehensive diagnostic evaluation outlined in the report, including laboratory tests, ophthalmological examination, and MRI findings, contributes to a deeper understanding of WD diagnosis and management. The inclusion of the “double panda sign” enriches existing literature on MRI findings, providing clinicians with additional diagnostic clues. Overall, this report not only presents a rare manifestation of WD but also underscores the relevance of imaging techniques, especially MRI, in elucidating the disease’s neurological aspects. Integrating clinical observations with radiological findings advances our understanding of WD and emphasizes the importance of a multidisciplinary diagnostic approach for optimal patient care.

WD is a rare genetic disorder caused by mutations in the *ATP7B *gene, which impairs hepatic copper transport, leading to systemic copper accumulation. The disease has an estimated incidence of 1 in 30,000 to 1 in 50,000 individuals worldwide, although this can vary based on geographic and ethnic factors [6]. It typically presents between the ages of 5 and 35, with late childhood, adolescence, or early adulthood accounting for most cases [7]. The clinical symptoms can vary in onset and presentation, affecting both sexes equally, although some studies suggest a slightly higher frequency in men. While WD occurs in people of all ethnic backgrounds, certain populations, such as Eastern European, Mediterranean, or Asian descent, can be more commonly affected due to genetic factors. The genetic basis of WD lies in mutations affecting the *ATP7B *gene, which encodes a P-type ATPase responsible for transporting copper across cell membranes, particularly in hepatocytes. These mutations lead to dysfunctional copper transport, accumulating copper primarily in the liver and CNS. Recent genomic investigations have identified a spectrum of *ATP7B* mutations, contributing to the variable phenotypic expression observed in WD [8]. WD exhibits a wide range of clinical manifestations, reflecting its multi-systemic nature. The classic WD triad includes hepatic, neurological, and psychiatric features. Hepatic dysfunction, often presenting with jaundice and hepatomegaly, is common, as are neurological symptoms such as tremors and dysarthria. Psychiatric symptoms, although less prevalent, may include mood disturbances and personality changes. Additionally, WD can present with associated features, such as Kayser-Fleischer rings in the cornea and hematological abnormalities, including coagulopathy and renal dysfunction due to copper deposition in the kidneys [9]. Understanding the locations of copper accumulation is crucial for comprehending the varied clinical presentations of WD. In the liver, copper accumulation can lead to hepatocellular apoptosis and necrosis, contributing to hepatic dysfunction. The basal ganglia is the predominant site of neurological symptoms in WD, with copper accumulation resulting in movement regulation issues. Copper accumulation in the cornea manifests as Kayser-Fleischer rings, observed during ophthalmological examinations [10].

Imaging, particularly MRI, is pivotal in diagnosing and monitoring WD, especially regarding neurological involvement. T2-weighted hyperintensities in the basal ganglia are a classic MRI feature of WD, indicating copper-associated lesions. Management of WD involves treating acute copper overload and instituting long-term maintenance therapy. Chelating compounds such as trientine and D-penicillamine facilitate copper excretion, while zinc supplementation inhibits copper absorption. Neurological status, liver function, and copper levels must all be routinely monitored. When hepatic dysfunction is severe, liver transplantation might be required [11]. Several disorders exhibit clinical features similar to WD, necessitating a comprehensive differential diagnosis. Hemochromatosis, for example, can manifest with hepatomegaly and hepatic dysfunction, but distinguishing features include iron overload and the absence of Kayser-Fleischer rings. Alcoholic liver disease and primary biliary cirrhosis share overlapping hepatic symptoms with WD, but distinct imaging features and histological findings help differentiate them [12].

## Conclusions

WD, although rare, presents a complex clinical picture requiring a comprehensive approach to diagnosis and management. This case of an early adolescent female underscores the importance of recognizing the triad of hepatic and neurological symptoms alongside associated features such as Kayser-Fleischer rings. Confirming neurological involvement mostly depends on imaging, especially MRI. Management strategies involving chelation therapy and long-term monitoring are essential for optimizing patient outcomes. A meticulous differential diagnosis is imperative in clinical practice to differentiate WD from conditions with overlapping features. Integrating recent advances in genomics and diagnostic modalities further enhances our ability to navigate the intricacies of WD.
